# Semiology of spontaneous generalized tonic‐clonic seizures in the epileptic baboon

**DOI:** 10.1002/epi4.12388

**Published:** 2020-03-20

**Authors:** Charles Ákos Szabó, David Andrés González, Sreekanth Koneru

**Affiliations:** ^1^ South Texas Comprehensive Epilepsy Center San Antonio Texas; ^2^ Department of Neurology UT Health San Antonio San Antonio Texas

**Keywords:** animals models, baboon, generalized tonic‐clonic seizures, Idiopathic Generalized Epilepsy, SUDEP

## Abstract

**Objective:**

The epileptic baboon provides an animal model for juvenile myoclonic epilepsy (JME), demonstrating spontaneous generalized tonic‐clonic seizures (GTCS) in addition to generalized myoclonic, absence and multifocal seizures. While photoconvulsive responses have been described in this model, spontaneous GTCS have not been characterized.

**Methods:**

In this study, we characterized 46 seizures in 7 epileptic baboons (5 females, 12 ± 3 years old) by video recording. While housed in single cages, the baboons were monitored for a median of 2 (range 1‐10) weeks, with high‐resolution, infrared‐capable camera systems. Each GTCS was evaluated for evidence of preconvulsive ictal symptoms, focal convulsive behaviors, duration of the preconvulsive and convulsive periods, postictal immobility, and recovery of an upright posture. The circadian pattern of GTCS was also for each baboon.

**Results:**

More than half of GTCS occurred in sleep, beginning from an upright position in all but one tethered baboon. Focal semiological findings were noted in 19 (41%) GTCS, and these included preconvulsive focal ictal motor behaviors as well as lateralized motor activity during the convulsions. The convulsive portion lasted 47 ± 10 seconds, whereas the entire seizure lasted 54 ± 21 seconds. Postictally, the baboons remained immobile for a median latency of 40 (range 14‐347) seconds, recovering an upright posture after 173 (range 71‐1980) seconds. GTCS demonstrated circadian patterns in all but one baboon, with 34 (74%) all seizures occurring between 1‐9 am.

**Significance:**

GTCS in the baboon revealed intersubject variability, but semiology remained stereotyped in a given baboon. Similar to GTCS in people with JME, focal symptoms were also observed in epileptic baboons. The postictal recovery period, characterized by postictal immobility and myoclonus as well as time to recumbency, also varied among baboons.

**Key Points**

- GTCS semiology varies among epileptic baboons, but is stereotyped in a given animal
- GTCS in epileptic baboons are similar to those in human IGE, both in duration and motor behavior
- Versive and rotatory behaviors frequently precede GTCS
- GTCS occurrence adheres to circadian patterns in most baboons
- Postictal immobility was interrupted by myoclonus in 5 baboons, possibly reflecting brainstem‐mediated activation

## 1 INTRODUCTION

For several decades, photosensitivity was the pathognomonic feature of epilepsy in the baboon; generalized myoclonic or generalized tonic‐clonic seizures (GTCS) could be provoked by intermittent light stimulation in the epileptic baboons.^1^, ^2^ These photoconvulsive responses were graded according to their somatotopic involvement and severity.^2^, ^3^ Allylglycine and pentylenetetrazol have also been used to trigger GTCS.^4^, ^5^ Although photosensitive baboons also experience spontaneous seizures, these were never systematically characterized.^3^ Spontaneous GTCS have been reported for several decades at the Southwest National Primate Research Center (SNPRC, Texas Biomedical Research Institute, San Antonio, Texas), which houses the largest captive baboon pedigree in the world.^6^ The veterinary staff typically identify epileptic baboons by observing convulsions, postictal lethargy, or by repeated craniofacial trauma due to seizure‐related falls.^6^, ^7^

Idiopathic generalized epilepsy (IGE) of the baboon was formally characterized in this colony, and closely resembles juvenile myoclonic epilepsy (JME).^6^, ^8^ The epileptic baboon demonstrates absence and myoclonic seizures in addition to GTCS, with onset in childhood or adolescence. On scalp EEG, the baboons have 4‐6 Hz generalized interictal spike‐and‐wave discharges, and demonstrate generalized ictal onsets.^8^ On intracranial EEG, the same baboons demonstrate both multifocal and generalized interictal epileptic discharges (IEDs), as well as focal or generalized ictal onsets.^9^ The epileptic baboons demonstrate normal development, neurological examinations, and essentially normal brain MRI and routine histopathology.^2^, ^5^, ^6^ The baboons respond to similar antiepileptic medications as people with JME,^4^, ^10^ although due to differences in hepatic metabolism, they may require different dosing for some medications to achieve therapeutic levels.^11^ Similar to people with epilepsy, spontaneous GTCS in baboons are also associated with earlier mortality due to sudden unexpected death in epilepsy (SUDEP).^12^

As a natural model for JME, as well as SUDEP, better characterization of spontaneous GTCS in the epileptic baboon will generate important clinical biomarkers for translational research. This study represents the first rigorous description of spontaneous GTCS in the epileptic baboon. The semiological features, including preconvulsive symptoms, GTCS duration, and focal convulsive symptoms will be evaluated to better understand phenotypic variability of GTCS and translatability of their primary IGE in humans. Furthermore, potential biomarkers for SUDEP, including postictal immobility (PI) and time to recumbency,^13^ and the relationship of GTCS to the sleep‐wake cycle, will also be evaluated.

## 2 METHODS

We identified seven baboons (B1‐B7, including 5 females, mean age 12 ± 3 years), who had undergone continuous video recording for a median of 2 (range 1‐10) weeks (Table 1). The baboons belong to a large, multigenerational pedigree housed at the SNPRC, but none of them were directly related.^4^ The incidence of GTCS in this colony is 25 per 1000 (2.5%) baboon years and their prevalence is 26%.^6^ All of the baboons were selected on the basis of seizures observed by caregivers. Scalp EEG studies, revealing generalized IEDs in six baboons (generalized rhythmical slowing in the remaining animal) and photoparoxysmal or photoconvulsive responses in 3 baboons. All but two baboons had a normal brain MRI (B3 had left‐sided colpocephaly, B7 had bilateral occipital horn elongation). All of but one baboon (B3) were euthanized due to recurrent seizure activity or seizure‐related injuries; B3 died suddenly and unexpectedly in his group cage, for no known cause (he had pulmonary edema and left ventricular hypertrophy on examination). All of the baboons had normal gross brain pathology on necropsy. The baboons were participating in three different funded studies at the time of their recordings: two baboons, B1 and B5, were recorded as part of an RNS implantation study,^14^ four baboons, B2‐B4 and B7, were recorded as part of a high‐frequency microburst VNS Therapy trial,^15^ and one baboon, B6, was implanted with intracranial depth, strip and grid electrodes.^9^ The differences between the protocols underlie the disparity of the recording times. The baboons were housed in single cages during the observation period, and GTCS were only included in the absence of neurostimulation or antiseizure medication therapies. The GTCS were recorded with cameras with infrared capability for nocturnal observation. Other seizure types, including myoclonic or absence seizures were not documented due to their subtle clinical presentation. All of the seizures were reviewed and classified by a board‐certified epileptologist (CÁS), while a second epileptologist (SK) independently reviewed seven GTCS recordings in five animals for the measurement of interrater reliability.

**TABLE 1:** Demographics and semiological features of GTCS in the baboon

Subject	Sex	Age (years)	# of GTCS	Study duration (weeks)	Scalp EEG (IED, PS)	Latency from “lights on” (seconds)	Preconvulsive State	Preconvulsive semiology	Duration of convulsion (seconds)	Lateralizing signs during convulsion	Median Duration Postictal Immobility (seconds)	Median Latency to Recumbency (seconds)
B1	M	10	6	2	N, N	NA	Sleep (N = 5) Awake (N = 1)	None	45 ± 5	L version (N = 1) R version (N = 1)	24 (N = 6)	124 (N = 3)
B2	F	16	8	10	Y, Y	189 ± 69 (N = 7)	Awake (N = 8)	Anticipated (N = 3); R head turning (N = 2)	50 ± 9	L version (N = 1) R version (N = 5) R clonic (N = 1)	35 (N = 7)	101 (N = 7)
B3	F	10	14	10	Y, Y	NA	Sleep (N = 14)	Left head turning (N = 1)	49 ± 5	L version (N = 2) R version (N = 1)	167 (N = 9)	186 (N = 9)
B4	F	16	8	5	Y, N	145 ± 7 (N = 3)	Awake (N = 4) Sleep (N = 4)	R before L rotation (N = 3)	49 ± 12	R version (N = 1)	25 (N = 6)	410 (N = 4)
B5	M	11	5	2	Y, N	396 ± 342 (N = 3)	Awake (N = 4) Sleep (N = 1)	R before L rotation (N = 5)	53 ± 18	None	56 (N = 4)	180 (N = 4)
B6	F	7	3	3	Y, Y	NA	Sleep (N = 3)	None	37 ± 6	L version (N = 1)	40 (N = 2)	107 (N = 2)
B7	F	12	2	1	Y, N	NA	Awake (N = 2)	None	38, 49	L version (N = 2)	49 (N = 2)	105 (N = 2)
Totals	5 F 2 M	Mean 12 ± 3	Total 46 Median 6 (range 2‐14)	Total 33 Median 3 (range 1‐10)	IEDs in 6 PS in 3	Mean 220 ± 182	Sleep 27 (59%)	Present in 14 (30%)	Mean 48 ± 10)	Present in 15 (33%)	Median 40 (range 14‐347) (N = 36)	Median 173 (range 71‐1980) (N = 35)

Note

B1‐7 baboon numbers, ± standard deviation, M(ale), F(emale), # or N number (when in brackets N refers to numbers of seizures), IED interictal epileptic discharges, PS photosensitivity, NA not applicable, R(ight), L(eft).

**FIGURE 1:**
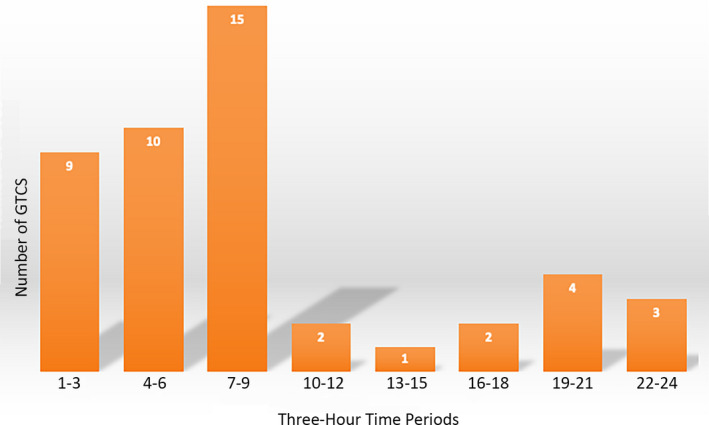
Diurnal variation of GTCS in 7 baboons. Legend: 24‐hour measurements are divided into 3‐hour blocks

The GTCS were classified according to whether they occurred from awake or sleep states based upon activity level, the latency of GTCS onset from the time the lights were turned on in the morning, presence of preconvulsive ictal behaviors; convulsive semiology and its duration as well as associated focal symptoms duration and semiology, specifically for any focal symptoms; and duration of postictal immobility (PI) and recovery of an upright posture, or time to recumbency. Sleep was determined by immobility for at least 30 seconds, either in an upright posture or, less frequently, in a prone position. The PI duration was adapted from a recent human manuscript,^13^ defined in our study as the time from the end of the clinical seizure (last clonic contraction) to the first detection of postictal nonrespiratory movement. the diurnal pattern for all seizure recurrences was plotted in a bar graph (Figure 1). Seven GTCS were selected from 5 baboons to evaluate interrater reliability of qualitative (occurring from awake vs sleep states, presence of preconvulsive symptomatology, PI) and quantitative (seizure onset, seizure duration, duration of convulsive symptoms duration, postictal immobility, and time to recumbency) markers, using interclass correlation coefficient scores. The studies were approved by the institutional animal care and use committees (IACUCS) at Texas Biomedical Research Institute and UT Health San Antonio, strictly adhering to all rules and regulations governing the use of laboratory animals, as outlined in the United States Public Health Service's *Guide for the Care and Use of Laboratory animals* ^16^ and the *Animal Welfare Act*.^17^

## 3 RESULTS

The results are summarized in Table 1 and Figure 1.

### 3.1 Semiology

Forty‐six GTCS were recorded, with median of 6 (range 2‐14) seizures recorded in each baboon. The median seizure frequency was 2 (range 1 to 3) per week. Twenty‐seven (59%) GTCS occurred from sleep in 4 baboons (B1, B3, B4, B6). In three baboons whose 14 seizures occurred postarousal (B2‐B4, B5), the mean latency of seizures occurring after lights were turned on in the morning (usually around 7 am) was 220 ± 182 seconds. All but two GTCS in one tethered baboon (B7) occurred in an upright position, regardless whether they were awake or asleep; B7 slept in a prone position due to instrumentation. A behavioral change was noted at the onset of 14 seizures in 5 baboons (B2, B4, B5, B7), lasting up to 30 seconds prior to the convulsive component, which included repeated versive head turning to one side or upwards (B2‐B4, B7), baboons lowering themselves to lowest point in cage (B2, B4, B5), and whole‐body rotatory movements (B4,B5) circling first to the right (3‐7 rotations) before the left (1‐3 rotations). The convulsive component of the seizures, characterized by clonic‐tonic‐clonic or tonic‐clonic motor activity, lasted 47 ± 10 seconds, but when including the nonconvulsive portion of the seizures, their mean duration became 54 ± 21 seconds. Focal features during the convulsive period included versive (8 right, 7 left) or focal clonic activity (3 right) in 15 (33%) GTCS, and occurred in all but one baboon. Combining lateralized preconvulsive and convulsive semiology, focal semiological findings were noted in 19 (41%) GTCS.

There was sufficient data regarding postictal immobility for 36 (78%) and time to recumbency in 35 (76%) GTCS. The missing data were due to accidental clipping of video footage during archival. PI lasted a median of 40 (range 14 to 347) seconds. PI was interrupted by either subtle movements after 9 (25%) GTCS, but by generalized, asynchronous myoclonus after 20 (56%) GTCS in 5 baboons (B1, B2, B4, B5, B7), The myoclonus occurred paroxysmally, recurring in some baboons until they were completely upright. The baboons were upright after median latency from the end of the clinical seizure of 173 (range 71‐2165) seconds. After three GTCS in B5 and one in B1, the baboons appeared to fall asleep, returning to an upright position only after 1296‐2165 seconds (22‐36 minutes).

### 3.2 Circadian pattern

Twenty‐six (57%) GTCS occurred nocturnally (lights were turned off between 7 pm and 7 am), with 19 GTCS occurring between 1 and 7 am (Figure 1). Nonetheless, GTCS peaked between 7 and 10 am, with 15 (33%) occurring within hours of awakening, and included the 14 seizures occurring shortly after lights were turned on in three baboons (B2, B4, B5). A circadian pattern was noted in 6 baboons, 7 (88%) GTCS occurring between 7 and 8 am in B2, 9 (64%) between midnight and 7 am in B3, 7 (88%) between 1 and 8 am in B4, 4 (80%) between 7 and 7:30 in B5, 3 (100%) between 7 and 9 pm in B6, and 2 (100%) occurring just after 8:30 am in B7.

### 3.3 Interrater reliability

Intraclass correlation coefficients (ICC) for discrete variables, such as onset from awake vs sleep states, presence or absence of preconvulsive head turning or body rotation, postictal immobility and myoclonus ranged between 0.77 to 1.0 (good‐to‐excellent reliability), while ICC for quantitative variables, including seizure onset, GTCS duration, duration of convulsive symptoms, postictal immobility, and time to recumbency ranged between 0.87 and 1.0 (excellent reliability).

## 4 DISCUSSION

Spontaneously occurring generalized tonic‐clonic seizures (GTCS) were characterized in epileptic baboons for the first time by this study using continuous video recording. While there was variability of seizure duration, preconvulsive and convulsive semiology, as well as time to recumbency between baboons, the symptoms were stereotyped and consistent in a given baboon. Regardless of whether or not the baboons were awake or asleep, almost all of the seizures were associated with a fall, generalized tonic‐clonic or clonic‐tonic‐clonic activity, and were followed by a period of immobility with subsequent recovery of an upright posture. The focal or multifocal character of the preconvulsive symptomatology, as well as lateralized motor symptoms during the convulsive period, confirmed the previous reporting of multifocal seizure onsets intracranial electrode recordings in epileptic baboon in this colony.^9^ Most of the GTCS occurred when lights were turned off (7 pm‐7 am) or within the three‐hour period after the lights were turned on (7 am) in the morning. Individual baboons tended to experience seizures either in wakefulness or sleep, but three experienced GTCS upon awakening, within minutes of the lights being turned on, and all but one baboon demonstrated a circadian pattern of the GTCS. These seizure patterns are typically reported in people with IGEs and, in particular, for JME.^18^, ^19^

There were several limitations of this study. We relied on single‐angle camera recordings of the baboons housed in single cages, and the baboons were frequently concealed behind the grating or metallic feeder. Outside light sources at times interfered with infrared lighting, and the resolution of the recording varied between cameras. Furthermore, there is still a need to record seizures in a larger sample, and simultaneous intracranial EEG and EMG recording would not only define electroclinical seizure onsets and termination, but also characterize focal behaviors and postictal recovery.

Due to the phylogenetic proximity and anatomical similarities between baboons and humans,^20^ it is not unexpected that primary GTCS in baboons would resemble GTCS in humans. Baboons demonstrate a similar duration of GTCS compared to people with IGE.^21^, ^22^ When including the preconvulsive part of the GTCS, the mean duration of 54 ± 21 seconds is only slightly shorter than the median duration of 66 (range 59‐75) seconds demonstrated in one human study, or the mean duration of 64 ± 28 seconds in a second study.^21^, ^22^ Both human studies were conducted in the epilepsy monitoring unit, where antiseizure medications are suddenly withdrawn, potentially resulting in more severe or prolonged seizure activity. As mentioned above, the baboons also demonstrated focal semiological features in 41% of their seizures, which mainly consisted of the preconvulsive symptoms, ranging from versive movements to rotatory behavior affecting the entire body, as well as convulsive symptoms, including version and/or asymmetric clonic activity. This is a similar prevalence to an epilepsy monitoring unit‐based study in people with IGE, 46% of whom demonstrated versive and focal motor activity, as well as “Figure 4” signs.^23^

In baboons, preconvulsive, versive semiology was identified in 4 baboons, two of whom also demonstrated subsequent rotatory behavior. Versive symptoms consisted of repeated head turning to one side, usually unaccompanied by other focal motor behaviors. Similar to versive seizures, focal rotatory seizures also tend to occur in the setting of focal epilepsy, but have also been reported associated with 3 Hz generalized spike‐and‐wave discharges in people with IGE,^24^ and can be followed by GTCS in people with JME.^25^, ^26^, ^27^ Hence, rotatory seizures may be clinical manifestations of preconvulsive, generalized ictal versive seizures may be generated by a parieto‐occipital discharge, but while the preconvulsive, versive symptoms may be generated by a parieto‐occipital discharge,^28^ whereas activation of frontal cortical‐subcortical networks has been implicated in the generation of rotatory behaviors.^24^, ^26^, ^27^

Postictal recovery patterns may provide quantifiable markers to help predict SUDEP, which also occurs naturally in the epileptic baboon.^12^ Postictal immobility (PI) was one clinical marker which was correlated with duration of postictal generalized suppression as well as respiratory dysfunction, both potential markers for SUDEP in humans.^13^ PI is thought to reflect seizure‐induced brainstem dysfunction. PI in the baboons was defined only clinically, as EEG was not simultaneously being recorded. Overall, PI is shorter in epileptic baboons (median 40, range 14 to 347 seconds) than in people with focal‐onset bilateral convulsive seizures (median 53, range −11 to 3156 seconds).^13^ Time to recumbency is another potential biomarker for postictal recovery. As baboons typically spend most of their lives upright, it appears recovery of the upright position is a natural response, perhaps even more as their survival from predators may have depended on it. Myoclonus exhibited in 5 baboons may serve arousal and recovery; due to its asynchronous, paroxysmal yet sustained appearance, while not affecting balance and tone as the baboons regain their upright posture, suggests that the myoclonus is likely to be subcortically or brainstem‐mediated, and not cortically generated.

Based upon the SeizureTracker database, circadian rhythms are commonly reported in a large cohort of people with epilepsy, and this phenomenon has also been confirmed by simultaneous intracranial EEG recording in a small group of patients enrolled in the NeuroVista study.^29^ The epileptic baboons also demonstrated circadian variability in seizure occurrence. Similar to people with JME,^18^, ^19^ baboons demonstrate a morning predominance of seizures, both before and after lights were turned on, and in some of the baboons, seizures were closely associated with awakening once the lights were turned on.

In summary, based upon the similar preconvulsive and convulsive semiologies, GTCS duration, PI and circadian seizure patterns, the epileptic baboon represents an excellent model for primary GTCS in humans.

## CONFLICT OF INTEREST

CÁS currently receives support from LivaNova for investigator‐initiated research, and he is a speaker for UCB Pharma. CÁS confirms that he has read the Journal's position on issues involved in ethical publication and affirms that this report is consistent with those guidelines.

## VIDEO ACCESS

Select videos of the GTCS in baboons B1‐B5 can be provided by the corresponding author (CÁS) upon request.
